# “Bringing you the best”: John Player & Sons, Cricket, and the Politics of Tobacco Sport Sponsorship in Britain, 1969-1986

**DOI:** 10.1163/26667711-bja10022

**Published:** 2022-08-11

**Authors:** Daniel O’Neill, Anna Greenwood

**Affiliations:** University of Nottingham, University Park, Nottingham, NG7 2RD, UK

**Keywords:** tobacco, smoking, cricket, sponsorship, advertising

## Abstract

This article explores some of the marketing strategies associated with the British tobacco industry’s sponsorship of sport during the 1960s and 1970s. It focuses on the British cigarette and tobacco manufacturer John Player & Sons and the firm’s pioneering initiative to sponsor one-day cricket, which began with the John Player League in 1969. The league was enormously popular and gained significant broadcast coverage, becoming an invaluable means of increasing public exposure for the company, in the context of the ban of cigarette advertising from British television. At a time when the link between smoking and disease was making headlines, John Player & Sons nimbly deflected attention away from the health issue, and instead consciously repositioned the tobacco company as a generous benefactor of the nation’s sport and leisure. Less conspicuously, but even more powerfully, spokespeople for the tobacco industry actively mobilised influential opinion behind the scenes in political circles. We show particularly how Denis Howell, Minister for Sport from 1964 to 1969 and from 1974 to 1979, became a valuable ally, acting as a bulwark against more restrictive government interventions into the sponsorship of sports by the tobacco industry. This alliance exposes changing industry–government relations and presents new historical context to better understand the way British tobacco manufacturers proactively sought to elide restrictions on their advertising activities from the 1980s onwards.

During the summer of 1970, the Medical Director of the Welsh Hospital Board’s Mass Radiography Service, Dr T. Francis Jarman, attended a John Player League match played by Glamorgan County Cricket Club at its Sophia Gardens ground. Objecting to the mismatch between the healthy pursuit of cricket and the less salubrious associations of this league’s commercial sponsor – the nationally popular cigarette manufacturer John Player and Son (JP&S) – Jarman signalled his concerns during the match’s tea interval by pacing slowly around the field’s perimeter wearing a sandwich board. On the front of the board was displayed the public health entreaty “Attend the Static Mass Radiography Unit for a Chest X-Ray” and emblazoned on the back – much more starkly – was the bold statement “Cigarettes Cause Lung Cancer.” Writing to the *Guardian* later in the year, Dr Jarman recalled that, after his understated and principled, solo march, he had then “settled down with an easier conscience to enjoy the rest of the game.”^1^

Whilst it received national press coverage, Jarman’s protest was a relatively rare one for the time. In fact, disquiet in Britain over the public health dangers raised through the sponsorship of sport by tobacco companies, although it sporadically caused controversy in both public and governmental circles, did not reach a critical boiling point until the mid-1980s. Even once attention was focused on the issue, although cricket was included in discussions, the sport remained relatively marginal in the heated debates that ensued; it was the TV coverage of tobacco-sponsored snooker and Formula One motor racing that became the prime focus for media investigations during the 1980s and 1990s. ^2^ Furthermore, when Jarman staged his protest in 1970, public health concerns over the ill effects of the explicit promotion of tobacco products had partly been assuaged by the 1965 ban of all television-based tobacco advertising. Perhaps precisely because sports sponsorship presented a newer, less direct, and more insidious marketing technique – the impacts of which were notoriously difficult to measure – it evaded formal regulation in Britain until 2002. It was only at this relatively late point that the UK-wide Tobacco Advertising and Promotion Act prohibited tobacco manufacturers from sponsoring any event, sporting or otherwise; a move which a few years later was echoed internationally by the 2005 World Health Organisation ban on all tobacco advertising, promotion, and sponsorship.^3^

While there has been much excellent research on the history of smoking in Britain, with some scholars even specifically focussing on the tobacco industry’s public relations and advertising strategies, the thorny relationship between the “unhealthy” tobacco sponsorship of “healthy” sport has received little attention by historians.^4^ Although the topic has been critically scrutinised by public health analysts on both sides of the Atlantic,^5^ the only sustained historical examination linking tobacco and sport has been a chapter within a book by Robert Proctor, discussing mainly American examples.^6^ The British history of sports sponsorship has, in the main, been tackled by sports historians who have unsurprisingly focused on describing what sponsorship meant for sport, rather than examining the motives of its sponsors or its impacts upon the wider public. Martin Polley, Dilwyn Porter, and Stephen Wagg, for example, have all traced the commercialisation of British sport in the decades following World War Two, albeit with each in slightly different sporting contexts.^7^ While excellent in terms of building a picture of the increasingly commercialist bent of sport over the course of the twentieth century, this scholarship does not address the uncomfortable public health issues deeply embedded within many sport sponsorship deals.

In an attempt to fill this gap, the present article centres on the case of JP&S, the hugely popular British tobacco company (est. 1877) owned by Imperial Tobacco, who specialised in affordable, mass-produced cigarettes.^8^ We focus on JP&S’s sponsorship of a new, fast paced, one-day cricket competition, known between 1969-1986 as the John Player League (JPL). Using the extensive, but rarely accessed, archives of JP&S held by Nottinghamshire Archives and Nottingham Museums – which contain a selection of business memoranda, advertising ephemera, and copies of the company’s in-house magazine – in combination with contemporaneous newspaper reports and government sources housed at the National Archives at Kew, we reveal a new story of how the British tobacco industry’s insidious influence operated. We show how, despite a growing awareness of the dangers to health posed by smoking, and despite (and also because of) legislation to ban tobacco advertising on television, the JPL presented an imaginative way for JP&S to circumvent public health moves designed to limit tobacco advertising while keeping its media presence high and maximising exposure for its company name. We show that the company did this through several avenues. By securing the naming rights to the League itself, JP&S cemented a business connection with cricket – the quintessentially pastoral national sport – to boost its corporate image by association. It moved attention away from the cancer connection and instead focused on the positive role the company played in reinvigorating the sport, not only for its mostly male match attendees but also through encouraging families to watch cricket together, either at matches or on Sunday afternoon television. At the matches themselves, we show how JP&S used additional techniques to promote their cigarettes. In particular, they employed female product demonstrators on match days to distribute free samples. These women were explicitly instructed by JP&S to ingratiate themselves with their male customers by using their supposed ‘feminine charms’ to promote cigarettes, while consciously avoiding any conversation about their products’ deleterious health effects.

Beyond the cricket field, however, another important part of the story is revealed through an examination of the tobacco industry’s behind-the-scenes political manoeuvring. Here, our arguments add another layer to Virginia Berridge’s powerful description of the changes which occurred in the relationship between the tobacco industry and government during the 1970s. Berridge shows how British tobacco companies were prepared to fulfil their (what we would now call) corporate social responsibilities, through the funding, from the 1950s onwards, of health research into smoking’s carcinogenic properties.^9^ This was part of a wider strategy, pursued jointly by the industry and government, to find ways to reduce the harm caused by tobacco products and to encourage safer smoking habits. To this end, tobacco manufacturers entered into voluntary agreements with the Department of Health and Social Security (DHSS) which covered ameliorative measures such as putting health warnings on cigarette packets and advertisements.^10^ Berridge describes, however, the breakdown of this cooperative relationship between public health interests and the industry during the 1970s, amidst a move away from policies predicated on harm reduction toward policies focusing on the elimination of smoking.^11^ Here we demonstrate how the tobacco industry, drawing upon the lessons learnt through the past advertising restrictions imposed upon it, consciously used a new backdoor route to influence policy and worked with interests which had been traditionally outside of the government’s smoking policy network. In short, the tobacco companies, including JP&S, cultivated a close relationship with the Minister for Sport, becoming key lobbyists for the extension of voluntary, rather than mandatory, controls to cover sponsorship. In these efforts, they were very successful: from 1977 to 2002, the regulatory landscape of sports sponsorship in Britain was characterised by voluntarism. Our insights add another layer to Berridge’s analysis of the relationship between government and the tobacco industry. We suggest that, during the 1970s, when the carcinogenic properties of tobacco were better known, the perceived growing misalignment between government policy and the industry’s interests was thought to be worrying enough for the tobacco industry that it stimulated some prominent members thereof to actively seek to mobilise government opinion in their favour. The industry’s hitherto obstinate strategy developed into overt petitioning efforts targeting political and public opinion, as the industry attempted to protect the favourable voluntary controls on sponsorship.

The tobacco industry’s pioneering use of sport sponsorship was a response to the broader smoking and health context. As Berridge has noted, smoking was one of the early issues around which a new style of public health developed in Britain from the 1960s onwards.^12^ With the apparent rise in chronic diseases such as lung cancer, and their link to behaviours such as smoking, public health became focused on getting individuals to take responsibility for their own health and encouraging people to make changes to their lifestyles. To these ends, bodies such as the Health Education Council (est. 1968), borrowing techniques from industry, began to run slick, advertising agency-created national publicity campaigns to encourage people to do things such as give up smoking and to drink in moderation.^13^ With traditional press and television advertising now featuring anti-as well as pro-smoking messages, the opportunity to associate cigarettes with healthy sports in a new and innovative way proved an attractive option for tobacco companies. As this article demonstrates, the sponsorship of sport obfuscated some of the public health initiatives taken to combat the promotion of smoking. The story of tobacco sponsorship often shows the industry working one-step ahead of public health organisations. It would be several years before a public health body took comparable action, such as when the West Midlands Regional Health Authority sponsored the West Bromwich Alboin football club during the mid-1980s and the team’s shirts featured a no-smoking symbol.^14^ In sponsoring cricket,British tobacco companies were also acting one-step ahead of their American counterparts, who did not begin to sponsor nationally televised sporting events until the early 1970s, and ahead of other comparable industries such as those producing and promoting alcohol.^15^ Breweries, unlike British tobacco, were more successful at exploiting football, with the names of various beer brands adorning football shirts from the 1980s onwards. Nevertheless, the alcohol industry, when sponsoring football, applied strategies honed by the tobacco industry: the renaming of trophies and competitions; taking advantage of sport to gain television exposure for brand and company names; and the at-scale placement of their products within male sporting and social spaces.^16^

After briefly outlining the wider context in which JP&S’s sponsorship of one-day Sunday league cricket evolved, the present article will show how, between the late 1960s and the late 1980s, the JPL provided opportunities for JP&S to mobilise public, sporting, and political opinion in its favour to serve as a bulwark against anti-smoking interests. We focus first on describing the creative marketing and advertising tactics used by JP&S to promote tobacco via cricket, before moving on to examine the company’s role in lobbying government sport representatives to pursue policies that supported the tobacco industry. This analysis extends, complexifies, and, to some extent, further darkens the history of the tobacco industry’s complicity in eliding and deflecting the harmful health effects of smoking (while being clever enough never to outright deny these effects) – an analysis which has previously concentrated on the American context.^17^

## 1 Cricket and cigarette advertising before the JPL

Historically, smoking has held a ubiquitous position within the homosocial culture which surrounded cricket. In 1884 and 1887, for example, members of the English and Australian national cricket teams took part in charity-matches in which they divided themselves into a team of smokers against a team of non-smokers. On both occasions, the smokers were outplayed by their non-smoking counterparts.^18^ Beginning in the second half of the nineteenth century, tobacco manufacturers reinforced the relationship between smoking and cricket through the marketing of their products. The company name, after John Player (who bought the business in 1877), made the sports connection seem an especially natural fit for this cigarette manufacturer in particular. Advertisements for JPS’s leading Navy Cut cigarettes featured idyllic, bucolic cricketing scenes and depicted W.G. Grace, who was perhaps the most well-known English cricketer of the era.^19^ The advertisements played directly into stereotypes of the sport being a gentlemanly, English game and emphasised the enjoyment and pleasure which could be obtained from both cricket and smoking.

Between the 1920s and 1950s, the popularity of both cricket and cigarettes soared. In 1920, 36,420 million cigarettes were sold in Britain and this number reached 99,560 million in 1956.^20^ By the end of this period, JP&S boasted the largest share of the British cigarette market, with sales of their brands accounting for around 39 per cent of all cigarettes sold in Britain.^21^ During this golden age, JP&S and its competitors actively developed and expanded their branding and advertising capabilities. One of their noteworthy tactics was to exploit the much-fêted cricketing rivalry between England and Australia. To this end, in the 1920s and 1930s JP&S reproduced portraits of the English and Australian cricket teams on two series of collectable cigarette cards included in packets of their product.^22^ Other manufacturers also incorporated themes of heroism, masculinity and celebrity into their advertising in order to capitalise on the excitement which this international cricketing rivalry generated. Army Club Cigarettes, for example, boasted that “The Australian Cricket Team Smoke Only Army Club[…] Men whose prowess depend upon eye nerve and wind smoke only Army Club Cigarettes” and leading English cricketer Jack Hobbs endorsed Sarony Cigarettes, declaring that there was “nothing throaty” about their smoke. ^23^ Shortly after the end of the Second World War, this trend continued and famous English cricketers Denis Compton, Len Hutton, and Godfrey Evans appeared in cinema advertisements for JP&S’s Medium Navy Cut cigarettes.^24^ The trio’s presence in the company’s marketing reflected a growing awareness on the part of leading English cricketers of their commercial value. For instance, during the 1950s, Hutton went on to also endorse Biro pens, Smiths Empire Watches, and, somewhat unscrupulously, Craven A cigarettes, one of JP&S’s competitors.^25^

By the late 1950s and early 1960s, however, cricket had nearly disappeared altogether from cigarette marketing. Instead, companies such as JP&S featured young couples and a heady romance theme in its advertising as it looked to exploit the growing teenage market.^26^ The disappearance of cricket from JP&S’s advertisements reflected the sport’s dwindling popularity. In 1946, 2.3 million had attended a first-class cricket match but this figure had dropped to just 700,000 by 1963.^27^ Cricket no longer had the attractive, well-liked image which was appealing to commercial interests and county cricket clubs faced an uncertain financial future. To address the sport’s perilous position, English cricket’s governing body, the Marylebone Cricket Club (MCC), set about reforming the domestic game and attempted to encourage “brighter” cricket which could stimulate the public’s interest.^28^ One-day cricket fulfilled this brief. It was faster paced and encouraged more aggressive, and therefore more entertaining, batting. After a trial tournament held in the Midlands, the MCC launched plans for a knock-out competition of one-day games made up by the first-class county clubs. In 1963, signing a deal worth only £6,500, the American razor blade company, Gillette (notably also a product, like tobacco, which underscored masculinity) became the competition’s first sponsor.^29^

The decision to launch a new competition to draw new crowds to the sport was part of a wider strategy towards updating cricket for the post-war age. ^30^ Furthermore, the move can be seen as an attempt by the MCC to more effectively exploit commercial interests for the whole of English first-class cricket’s benefit, rather than having leading cricketers monopolise advertising revenue as under the earlier endorsement model. The prolific product endorsement achieved by certain cricketers during the 1950s was not repeated by later generations during the 1960s and 1970s.

Whilst cricket’s desire to update its staid image for the affluent post-war world encouraged the sport to exploit new commercial opportunities, the tobacco industry also had to face up to a cold new reality during the 1950s and beyond. After Richard Doll and Bradford Hill’s famous *BMJ* paper,^31^ the noise correlating cigarette smoking with increased rates of lung cancer became progressively louder.^32^ Although the tobacco industry and some elements of the media insisted that no direct causative correlation linked smoking to cancer,^33^ manufacturers quickly realised that they had to work out strategies to mitigate the sales problems posed by the growingly negative associations between smoking and health. In 1956, in an important market research study produced for Imperial Tobacco (JP&S’s parent company) and distributed to the advertising agencies with which JP&S worked, the market research organisation Research Services Limited (RSL) recommended that cigarette advertising should attempt to recruit to the smoking habit young people aged roughly between 15 and 23. On the smoking and health issue, RSL concluded that, generally: “[v]ery little can be done on a mass scale to quieten people’s fears about their health” but advised that advertisements should show that “those most dependent on good health (e.g. sports figures and men who perform physically strenuous jobs) and quite simply, healthy-looking individuals are very frequently, smokers themselves.”^34^

Such recommendations help explain JP&S’s turn towards featuring attractive young couples in their advertisements, but such imagery was featured only fleetingly, as the advertising environment became constricted and the smoking and health controversy deepened during the 1960s. In 1962, the Royal College of Physicians published their watershed report *Smoking and Health* which garnered widespread attention in the press and sold 20,000 copies in six weeks.^35^ The report urged the government to adopt restrictions on advertising.^36^ In a move which foreshadowed the industry’s voluntary response to proposed restrictions on sponsorship during the 1970s, British tobacco manufacturers, represented by the Tobacco Advisory Committee, agreed to follow a code which forbade certain appeals and images appearing in their advertisements. The code was produced by the Independent Television Authority (ITA) and it asked manufacturers to avoid several “areas of danger” in their advertisements, including “‘Hero appeal’ and the appeal to ‘manliness’.” Supplementary advice produced by the copy committee of the Independent Television Companies Association for advertising agencies with cigarette manufacturers on their books, requested that agencies take “special care” when featuring “characters outside the orbit of ordinary life and likely to inspire admiration and emulation.” The advice clarified exactly the kind of imagery that would be problematic if featured in a cigarette advertisement with an example: “people of extreme distinction, sophistication or authority should be avoided. A cricketer leaving a village cricket pitch is unlikely to cause any problem, but a cricketer leaving Lord’s after scoring a century would be unacceptable.”^37^ Later in the year, the advertising code was voluntarily extended by the tobacco industry to cover press, poster, cinema and radio advertisements.^38^ After this point, images of sports people tended to feature far less frequently, but they did not by any means disappear from British cigarette advertisements.

The most dramatic change to the cigarette advertising landscape occurred, however, in August 1965, when cigarette advertisements were banned from British television. Since its launch in 1955, commercial television had quickly become the main advertising medium for the British tobacco industry. Collectively, manufacturers spent just under £7 million on television advertisements in 1964 and an estimated 2,000 cigarette advertisements were broadcast each month during the first half of 1965, before the ban came into effect.^39^ The ban came after repeated calls for such a move from Labour MPs and the move did not require legislation since the Postmaster General, the MP Tony Benn, had the power to compel the ITA to refrain from broadcasting certain material.^40^

## 2 The birth of the JPL

It is against this shifting backdrop that JP&S and its advertising agencies started to look again at sport for new ways of getting its brand into the public eye, in a positive way that maximised exposure without causing moral outrage. The balance was a difficult one to strike, as the public health risks of smoking were very much in the public eye. By 1965, despite an expansion in the overall cigarette market during that decade, JP&S only had a 24 per cent share of the market, having been overtaken by its main competitors, with Wills of Bristol holding 36 per cent and Gallaher of Belfast 33 per cent of the market.^41^ JP&S’s waning fortunes were strongly connected to its inability to shed its reputation for manufacturing plain (unfiltered), strong-tasting cigarettes.^42^ Due to the prevailing health concerns and the growth in price of cigarettes due to increases in the tax on tobacco during the 1960s, smokers were increasingly smoking brands which were shorter, cheaper and filtered.^43^ Up until the mid-1960s, JP&S struggled to launch a successful filter brand but this changed in 1966 with the introduction of a new cigarette, Player’s No. 6. Denied television advertising, JP&S carved out a unique position for their new brand; at the time of its launch and for its first few years, Player’s No. 6 was the only cigarette in the then cheapest price bracket to offer smokers a gift scheme.^44^ A promotion technique pioneered during the interwar years, gift schemes offered smokers the opportunity to save up coupons included in packets of cigarettes, which could be exchanged for promotional gifts.

In another novel move, to gain publicity for Player’s No. 6, the company sponsored and associated the brand with a wide array of leisure, entertainment, and sporting events. Although JP&S had previously run promotions at dance halls, Butlin’s holiday camps, and in shops, the company started to place greater emphasis on so-called “below the line” marketing after the television advert ban. The company set up a “sales promotions unit” (later renamed the “special events unit”) and employed a dedicated team of regionallybased representatives whose role it was to put on promotions in support of JP&S brands, particularly Player’s No. 6.^45^ This activity included sponsoring a diverse range of sports, including those which were relatively niche such as canoeing, autocross, and hovercrafting. Facilitated by affluence, technology, shorter working hours and the growth in car ownership, the 1960s saw a proliferation of new pastimes and an increasingly crowded commercial leisure market. As JP&S’s then marketing director Geoffrey Kent put it, the company liked “to be associated with new ideas, new happenings, excitement and success.”^46^ By sponsoring activities which could impart to its products an air of trendiness and excitement, and by implication a certain amount of wholesome healthiness, the company could position itself as a forward-thinking benefactor of the latest leisure trends.

In the late 1960s, however, JP&S’s sponsorship strategy changed. A manager at the company acknowledged that the specialist sports which JP&S was supporting could only provide “[t]actical deep penetration to limited audiences” rather than “national mass coverage” which was more desirable.^47^ JP&S stopped its support for more unusual activities such as hovercrafting, a venture which a company spokesman rather sheepishly admitted was “probably a few years ahead of its time,”^48^ and began sponsoring more high-profile activities, such as the Formula One racing team, Team Lotus. Marketing objectives set for the Player’s No. 6 brand from 1968 reflected this change in approach. Promotions were now intended to exploit “established events to obtain quality endorsement rub-off.”^49^ Such aims were reflected in the objectives JP&S had for its cricket league: “[t]o enhance the image of John Player & Sons, and of Player’s No. 6, by association with a prestigious modern and successful National Sporting Activity and to be seen to be helpful to that activity.”^50^

In cricket, JP&S found a sport eager to make commercial connections. A precedent for the sport to associate itself with non-sporting products had already been set by Gillette’s 1963 sponsorship of one day cricket, but of even greater relevance were moves made by rival tobacco manufacturer Rothmans, owned by the Rembrandt Tobacco Group from South Africa. Rothmans supported cricket in England from 1963 onwards, and enjoyed great success through its backing for the International Cavaliers team. The Cavaliers were the brainchild of enterprising sport agent Bagenal Harvey (who became the chairman of the club) and four of his clients, the English cricketers Denis Compton (the club president),Godfrey Evans, Ted Dexter and Colin Ingleby-Mackenzie.^51^ The Cavaliers team was made up of famous former English cricketers and current international players, and the team staged one-day benefit matches with various English county sides on Sunday afternoons. In order to comply with the Sunday Observance Laws, spectators were allowed in for free, or in exchange for the purchase of a raffle ticket or match card.^52^ From 1965, Cavalier matches were televised on BBC2, with broadcasts beginning at 2:30pm so as not to impinge upon church attendance.^53^ Rothmans would also provide financial support for international teams’ tours of England, so long as their leading players were available for Cavaliers games.^54^ The Cavaliers’ Sunday matches proved immensely popular, with 130,000 people turning out to see them during the 21 matches in which they took part in 1968.^55^

The development and popularity of the International Cavaliers had been looked upon with envy by the MCC. In response, the governing body in 1968 launched its own Sunday matches, announcing its intention to sponsor a one-day cricket league. JP&S successfully bid for the endorsement of the new league, paying the MCC £67,174 for the privilege of sponsoring the League’s first year. This included £35,000 to be divided amongst the seventeen first-class counties taking part in the league, and £19,448 to cover the match fees of the cricketers taking part in the league. Each player was paid £6.10.0 per appearance.^56^ With the new league, the MCC was trying to establish its own monopoly on Sunday cricket and to capitalise on the popularity of the Cavaliers’ brand of one-day cricket. The MCC forbade first-class county cricketers from participating in any televised Sunday cricket other than its new league and it agreed with the BBC that its Sunday games would take precisely the television slot previously occupied by Cavalier matches.^57^ In addition, the MCC’s secretary Billy Griffiths rejected the idea that the Cavaliers could participate in the new Sunday league, citing as justification the clash of sponsors.^58^ Rothmans offered the MCC £40,000 for the Cavaliers to play televised matches against the English counties on those ‘spare’ Sundays when league matches were not scheduled, but this was rejected.^59^ Rothmans complained that the MCC had communicated to them that JP&S was to be the only tobacco company allowed to sponsor televised cricket.^60^ The Cavaliers continued to play, albeit more sporadically, and with ITV broadcasting their matches, but this finally stopped in 1970, the same year that Rothmans ceased supporting the team.^61^ JP&S used sponsorship to build partnerships with sports in which the company’s presence was “unique, distinctive and dominating,” and the exclusion of Rothmans from Sunday cricket reflects how the competitiveness of the cigarette market spread to cricket via tobacco sponsorship.^62^

## 3 How the JPL advanced JP&S

The JPL worked for JP&S in three distinct ways. First, it provided the tobacco company with TV airtime on a scale not easily accessed since the 1965 TV advertising ban; secondly, it allowed for the development and refinement of a surreptitious publicity technique which lodged the company generally within the public consciousness and associated it with wholesome, healthy entertainment, while lessening explicit references to more publicly recognised “unhealthy” cigarette brand names; and thirdly, the League matches created new physical promotion opportunities, which played on gender stereotypes through the employment of attractive product demonstrators to distribute free samples.

Turning first to the television exposure that the arrangement allowed, one of the most important aspects of the JPL for its sponsors was that it guaranteed the company extensive television exposure, with BBC2 regularly broadcasting games. During a typical season, 136 one-day matches took place between the 17 first-class county sides in England and Wales, with 17 of these being broadcast by BBC2.^63^ During the first season, average television viewer figures indicated 1.25 million people watched each match.^64^ Average viewing figures had risen to 3.2 million per match by the 1975 season, and JP&S calculated that the JPL received 96 hours of television coverage during the 1976 season, making it one of the company’s most economical events in terms of measuring exposure in relation to financial outlay.^65^ When looking to cut down expenditure on sport sponsorship in the late 1970s when the financial outlook for the British tobacco industry worsened, JP&S’s managers indicated that the League should be the last event to go.^66^ The company clearly valued the regular and stable television coverage the league offered at a relatively low cost. In comparison, JP&S spent far larger sums on sponsoring Team Lotus. For instance, JP&S paid the Test and County Cricket Board (TCCB, which had been set up to oversee domestic cricket by the MCC) £335,000 to sponsor the JPL between 1976 and 1978.^67^ Whereas, for the same period, the company paid Team Lotus £1.35 million to sponsor its racing cars.^68^

The JPL therefore offered JP&S access to television audiences at a time when advertising options were restricted and routinely threatened. For JP&S, sponsorship became justifiable in terms of “commercial considerations, i.e. television coverage, audience size and sales opportunities” alone.^69^ This change was prompted by the political scrutiny to which cigarette marketing was subject because of its negative health effects. JP&S saw cricket sponsorship as its “insurance premium” and “an assurance against further media restrictions when fewer, if any, conventional media will be available.”^70^

The JPL moreover allowed JP&S to expand awareness of the company name in a subtle new way that was much less likely to cause public, or political, offence at a time when smoking was becoming more and more associated with cancer. Jim Shaw, in the late 1960s, described the benefits of sponsorship as being one of image transfer, where the “excitement, the newness and the success” of the sponsored sport “are spread to products by creating a sympathetic and emotional understanding between the brand under promotion and the smokers.”^71^ It was understood that, unlike advertising, sponsorship worked in a less direct manner, conferring benefits which were, as described by one JP&S manager in 1975, of an “intangible nature and difficult to assess.”^72^ Despite this, however, experience quickly led JP&S to understand that it was, in certain ways, more advantageous for them to use their more remote “house” name (Player’s or John Player) rather than their cigarette brand name (Player’s No.6).

**Figure 1:**
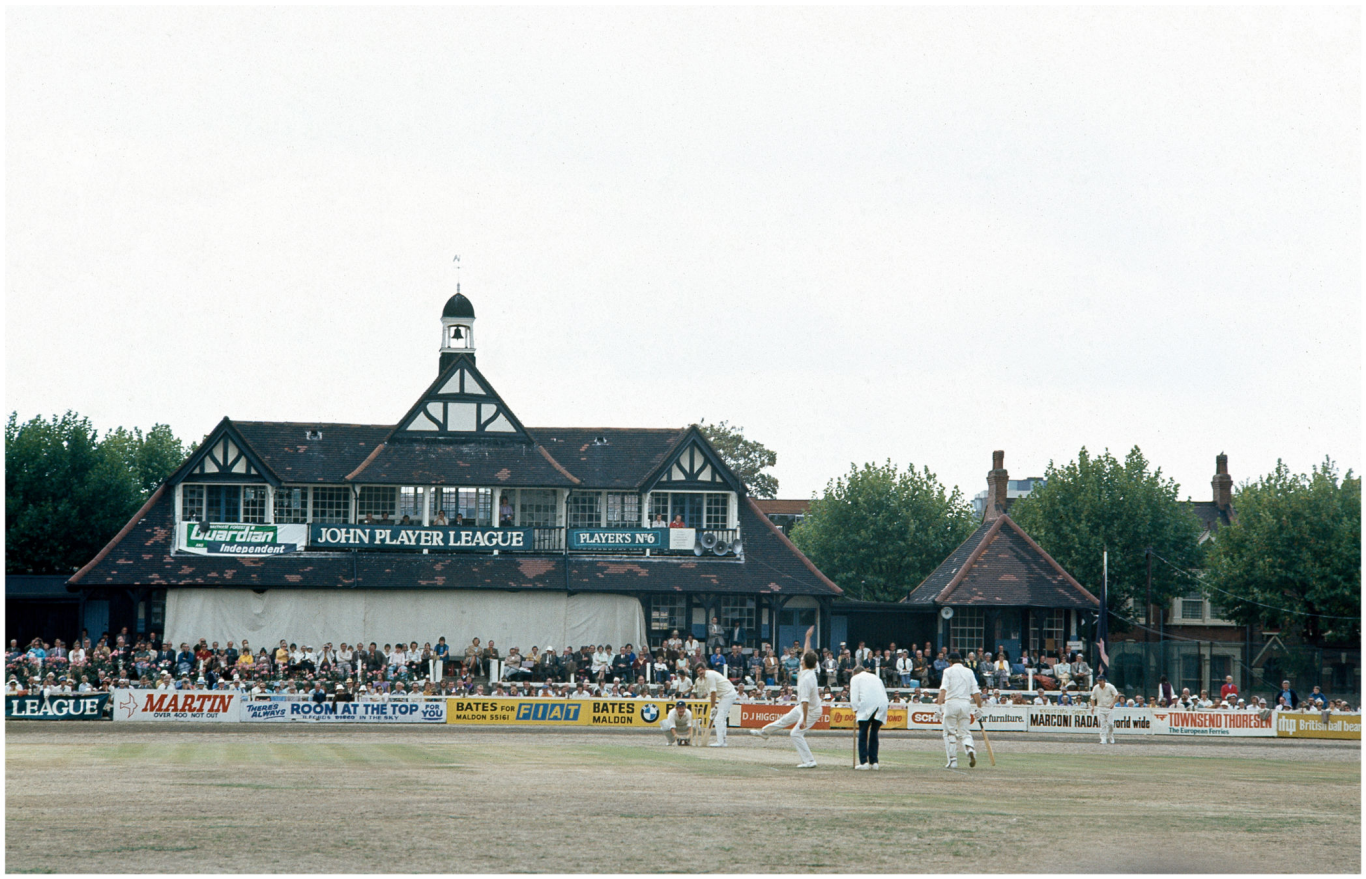
John Player League match between Essex and Yorkshire at Leyton, ca. 1975. Credit: Bill Smith/Getty Images.

Initially, JP&S had wanted the league to be called the “Player’s No. 6 County Championship.”^73^ This proposal was abandoned, partly because the MCC thought it was too similar to the title of the traditional domestic cricket league, the County Championship, and also because of what JP&S’s management called “strategic reasons.”^74^ Manufacturers’ use of gift schemes to promote cigarettes was a contentious political issue during the late 1960s. In October 1967, the Labour Minister for Health, Kenneth Robinson, made a statement in the House of Commons announcing his intention to “introduce legislation in due course to take powers to ban coupon gift schemes.”^75^ JP&S did not wish to provoke further political reaction and so moved away from closely identifying the league with its coupon brand, Player’s No. 6. Thus, the competition during its first season went under the name “The Player’s County League.” This title was also not without its problems. A National Opinion Poll (NOP) conducted in the summer of 1969 revealed that 75 per cent of the public did not know that the “Player’s” in the League’s title referred to JP&S. This is where the ambiguous brand name worked against corporate interests, as twice as many people believed that the league was run by cricket players as opposed to JP&S.^76^ To rectify this, for its second season and beyond, the league became known as “The John Player League” (see Fig. 1).

Settling on a less brand-specific and a more company-orientated league name had certain advantages, even if it occurred more as a pragmatic response to circumstances, rather than as a result of prudent strategic design. For one, the new name helped to ensure media outlets would unambiguously identify the competition as being sponsored by JP&S.^77^ JP&S’s managers pressed the MCC to do all they could to ensure that press and television presenters identified the league properly, with MCC secretary Billy Griffith agreeing he would try to “persuade people gently.”^78^ In addition, the BBC was wary about giving publicity to any brand names in its broadcasts. Initially, the broadcaster allowed only two JP&S banners to be in view of its cameras.^79^ In 1972, the BBC and the Independent Television Authority agreed with UK tobacco manufacturers that any brand advertisements or banners which were in camera shot at sporting events could be covered up by television crews covering the event.^80^ Banners which mentioned only company ‘house’ names, as opposed to cigarette brand names, were exempt from this – so too were cigar or pipe tobacco brand names. So JP&S complied with the regulations by using signs during televised cricket matches which simply bore the text, ‘John Player League.’^81^

In respect to using “John Player” in its title, the cricket league reflected a general marketing strategy which JP&S developed and pursued during the 1970s, whereby virtually all events sponsored by the company included “John Player” in their name. For instance, the British Grand Prix became the John Player Grand Prix between 1972 and 1976. Managers’ believed that using the ‘house’ name in the title of events would be “less politically contentious” and “more readily acceptable by media coverage, particularly television”.^82^ By the mid 1970s, JP&S understood sponsorship to be a “communication medium” and “[a] way of modifying our house image,” with the company looking to use an association with health-giving sport as a means of moving away from its earlier reputation for manufacturing “Plain – i.e. strong – cigarettes.”^83^ Instead, deflecting public and political attention away from health concerns, the company looked for events which could help project “John Player & Sons as a modern, dynamic, progressive and popular company.”^84^

By the 1970s, this gentler form of advertising which attached JP&S to prestigious national sporting events was firmly established. This strategy is best represented by a unique public relations campaign which JP&S embarked upon in the early-1970s. Between 1972 and 1974, JP&S placed double-page advertisements in colour supplements such as *The Sunday Times Magazine*. The advertisements did not mention any cigarette brands or even the fact that JP&S was a tobacco company. As a result, the advertisements did not have to carry a health warning, which had become mandatory for all cigarette advertisements which appeared in the press from 1971 onwards.^85^ The advertisements ran during the summer months and each provided information on a different JP&S sponsored event, such as the JPL, or other well-regarded events which the company did not sponsor, such as the Edinburgh Tattoo, Wimbledon or the Southport Flower Show.^86^ Alongside these event profiles, the advertisements featured a “seasonal calendar presented by John Player” which provided a useful diary of upcoming sports and arts events. It was a clever move. The spreads featured the tagline “John Player bringing you the best” or (in the case of non-JP&S sponsored events) “John Player bringing you news of the best.”^87^ In support of the campaign, JP&S set up an information bureau at premises on Oxford Street, London. The bureau was equipped with an information desk, a library of reference books, and staff trained by the English Tourist Board.^88^ JP&S billed this as “a centralised information service,” to which the public were invited to telephone, write, or visit. JP&S crowed that “staff will make every effort to answer any question you may have on any event – and also offer suggestions for outings.”^89^

This “bringing you the best” campaign distanced the company from its products and instead attempted to enhance JP&S’s reputation, with the campaign helping to fulfil a broader marketing objective for the company. In emphasising the support JP&S was providing for the nation’s sporting and cultural life, the company could “[a]ppear to be helping Britain.”^90^ The unbranded nature of the advertisements also allowed JP&S to appeal to families, something which would not have been permitted in traditional tobacco advertising. The campaign encouraged the public to “[t]ake the family to see one of the really enjoyable games in the John Player League this Sunday.”^91^ Far from distancing the company name from its products, however, sponsorship helped gain publicity and goodwill for the name ‘John Player.’ During the 1970s, JP&S launched a series of new cigarettes which featured the John Player name: John Player Special, launched in 1971; John Player Kings, launched in 1974; and John Player King Size, launched in 1976. There was a malleability to the name ‘John Player’ which allowed it to be both a company name, suitable for sponsorship and advertisements without health warnings, and a brand.^92^

The JPL was designed to foster excitement, with JP&S looking to promote its name through a “lively and invigorating atmosphere.”^93^ JP&S attempted to encourage thrilling play by providing two pots of £1,000 prize money per season which was to be shared out between all players who either hit a six (the maximum number of runs which can be scored off one ball) or who bowled four or more wickets during a match.^94^ JP&S believed that people were more receptive to advertising and promotions when they were enjoying their leisure time. Jim Shaw, JP&S’s Promotions Manager, emphasised this when outlining the company’s “Promotional Policy”:

Clearly, it makes a great deal of sense to communicate, in a friendly way, when they are enjoying themselves. After all, cigarettes are enjoyed in sociable surroundings and sporting events which capture the interest of the vast majority of the population of this country are an ideal setting for our message.^95^

**Figure 2:**
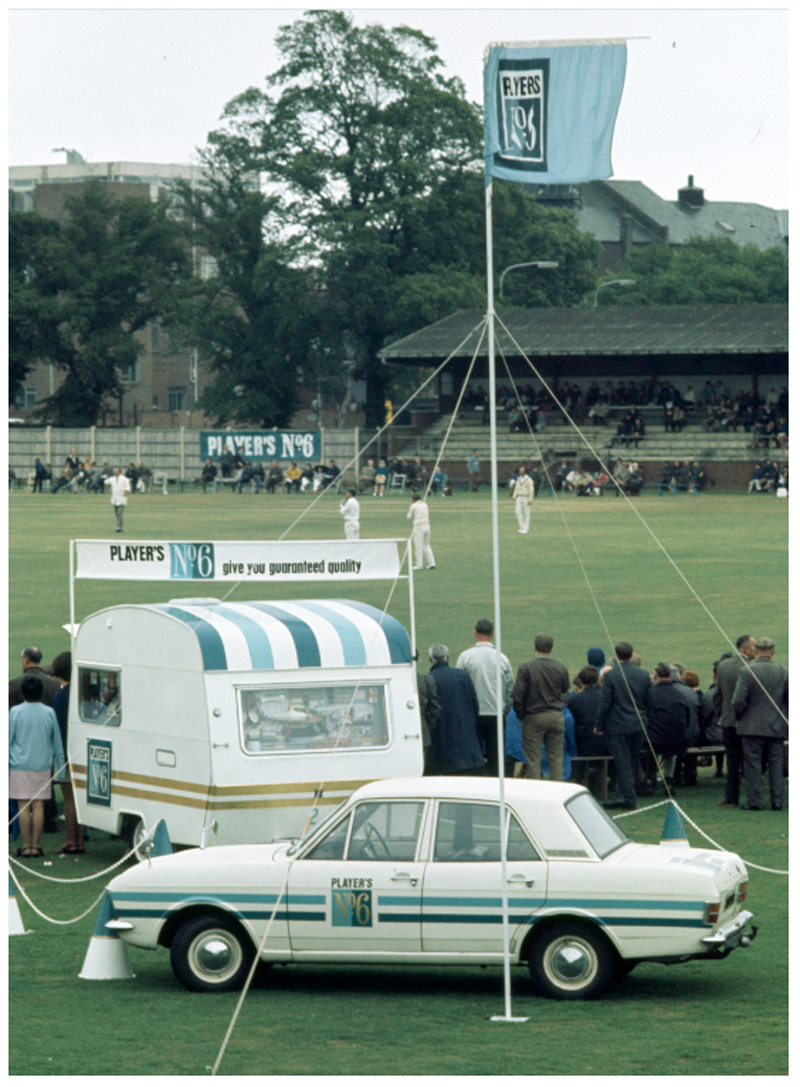
Player’s No. 6 kiosk at the United Services Ground, Portsmouth, 19 July 1970. Credit: Patrick Eager/Getty Images.

**Figure 3:**
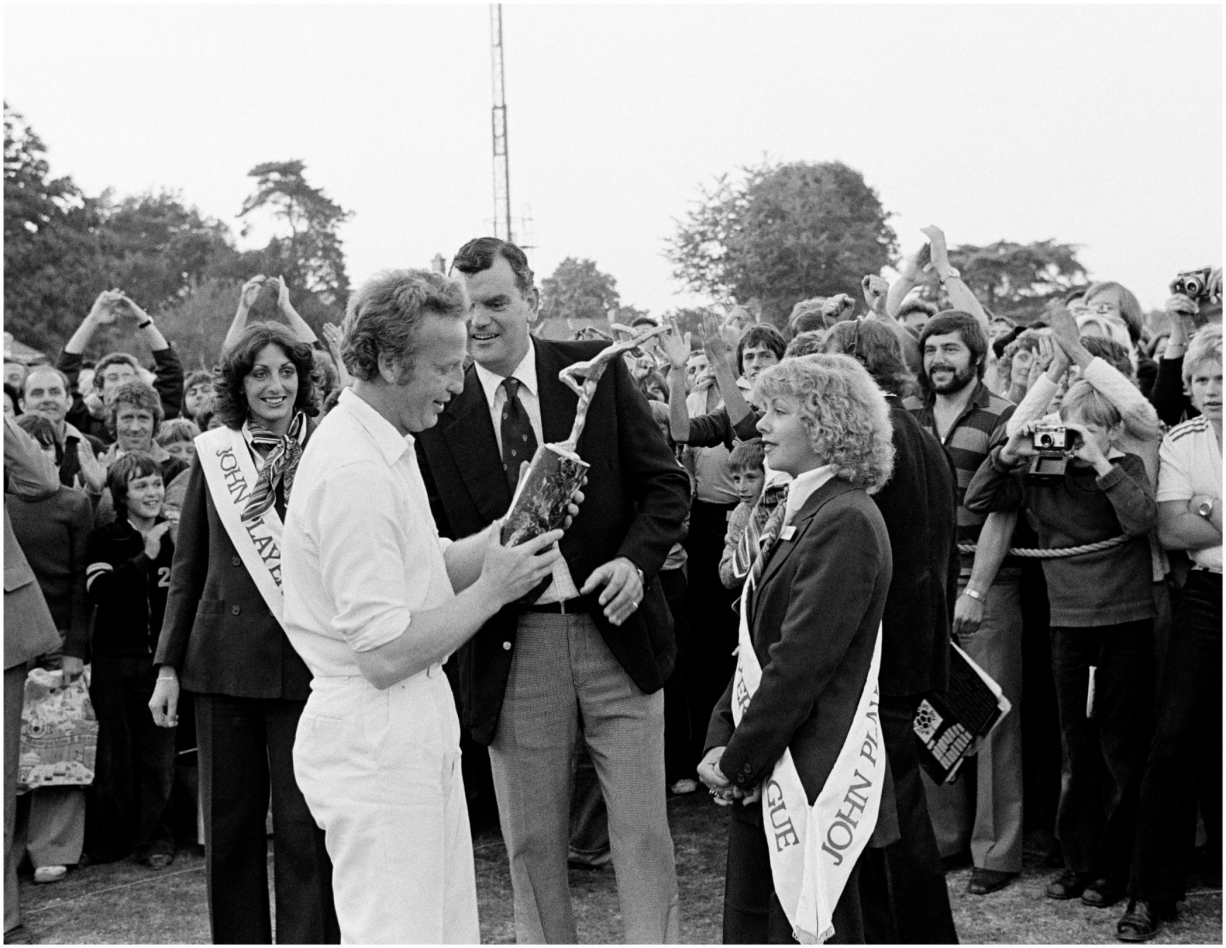
Hampshire captain Richard Gilliat being presented with the John Player League Trophy by two female demonstrators and the BBC commentator Jim Laker, at Dean Park, Bournemouth, 3 September 1978. Credit: Patrick Eager/Getty Images.

The company also promoted its products intensively at JPL matches. Particularly, they recruited teams of female demonstrators who were to be the human points of contact between the company and consumers at events. Demonstrators were to move around the crowds of watching spectators selling and giving away free samples of cigarettes and were to staff the mobile Player’s No. 6 kiosk which was set up at grounds on match days (see Fig. 2). In addition, these women were employed to help JP&S keep important stakeholders happy and to ensure the smooth running of League matches. If asked to do so, they were to have lunch with the two teams, providing the captain of each team, and the hosting club’s secretary, with a free packet of Player’s No. 6. At televised matches, moreover, they were instructed to visit BBC producers and cameramen in order to “create a climate of good will.” As if that wasn’t enough, the demonstrators were to act also as walking advertisements, wearing uniforms which repeated the liveries and colours of Player’s No. 6. Alongside any VIPs present on the day, demonstrators were on hand to present a cheque of £50 to the winning team (see Fig. 3). JP&S hoped the prizegiving could take place as the teams left the field at the end of the match, in order for television cameras to pick up the exchange. These employees were also told to bring out drinks for the players on hot days.^96^

JP&S looked to recruit demonstrators who were young and attractive, with the company employing a narrow and prescriptive definition of feminine beauty. Job advertisements for the roles stressed that applicants should be of “attractive appearance and sparkling personality,” be aged between 20 and 24, have “considerable selfconfidence,” be single, be between five feet four inches and five feet eight inches, and have “a high degree of intelligence and a persuasive but pleasant manner together with organising ability.”^97^ In 1969, demonstrators were paid £5 per match.^98^ Their presence contributed to a clear gender dynamic at work at JPL matches: women were used to sell cigarettes to men. During the first season of the League in 1969, JP&S’s market research revealed that around 90 per cent of those attending JPL matches were male.^99^ As the job advertisements attest, JP&S had a specific feminine role in mind for their demonstrators. The requirement for demonstrators to be single perhaps reflected the company’s traditional notion of the age-bracket wherein it was deemed appropriate for a woman to work, but there was also an expectation that demonstrators would use their ‘feminine charm’ to attract customers to JP&S’s products and potentially to be willing to engage with important stakeholders at matches in a lightly flirtatious way (if that was what the situation ‘demanded’), as the euphemistic instruction that they should “create a climate of good will” suggests. Although it was hoped that their presence at matches would help to foster an enjoyable atmosphere, female demonstrators’ promotional work was not always welcomed by spectators. A JP&S representative complained that the “audience [were] annoyed at girls interrupting their cricket by sampling and selling” at one of Nottinghamshire’s home games during the 1969 season.^100^

The initiative was not exclusive to JP&S; other cigarette companies also utilised the services of female demonstrators to promote their products at matches. Sometimes, this caused controversy. For example, in May 1976, *The Sunday Times* reported that “cigarettes are offered more or less indiscriminately” at cricket matches, and published a picture of a Gallaher demonstrator offering free cigarettes to “a group of fresh-faced youths, coltish and lanky” at one of the company’s Benson & Hedges Cup matches.^101^ The report caused something of a furore at the Department for Health and Social Security (DHSS), with Gallaher having to deny the claims and send the government department its guidelines on sampling. Gallaher’s policy was that free samples should only be given to those aged 18 and over and they declared that they were, by 1976, instructing their demonstrators about what to do when they were “unavoidably drawn into discussions on smoking and health.” Gallaher stated that “Under no circumstances should you raise your own personal point of view” and questions should be answered with a cursory “I’m sorry, but I do not know.”^102^ Similarly, JP&S’s demonstrators were under strict instructions not to give out free samples to anyone they thought to be aged under 21, although this age appears to have later dropped to 18.^103^ Even while they were expected to be vigilant in these interactions, the JP&S’s demonstrators themselves were also under scrutiny. One manager instructed demonstrators to “Think and speak well of John Player & Sons at all times” and “speak clearly, smile and always remain courteous however adverse the circumstances may be.”^104^ These instructions were not always followed, however: the same manager lamented that “It chills the spine to hear audiences being exhorted to ‘smoke themselves to death’ – this has happened in the past.”^105^

## 4 JP&S and the politics of sports sponsorship

The scrutiny that demonstrators came under reflected the increasingly hostile political environment which the British tobacco industry found itself navigating from the early 1960s, as the public health risks of smoking became more widely accepted. It is significant that sponsorship agreements managed to escape, for another dozen years, restrictions comparable to those that had been imposed on television advertising. This is not to say, however, that there was no criticism of sponsorship arrangements between sports and the tobacco industry.

In 1970 the Sports Council, which was a government advisory body set up in 1965 (but made independent in 1970) and whose role it was to help develop sport in Britain, launched an enquiry into sport sponsorship. Its report, published in 1972, argued that “acceptance of sponsorship by a governing body implies approval if not full endorsement of the kind of product the sponsor manufacturers or sells.” It recommended that the government “give positive guidance about a code of practice when cigarette companies sponsor sport, before the involvement in sponsorship by cigarette manufacturers, already greater than other sponsorship, increases still further.”^106^ However, the DHSS, under the Conservative Minister of Health Sir. Keith Joseph, was reluctant to act, for fear of appearing to be “spoilsports.”^107^ A civil servant highlighted the JPL as a clear example of the tobacco industry gaining publicity for its brands in spite of the TV ban, but warned his colleagues that “it would be bad for our image to be seen to be stopping sports sponsorship.”^108^

It was not until Dr David Owen became the Minister for Health in 1974, as part of a Labour government, that moves were made against tobacco sponsorship, albeit only suggesting voluntary compliance. At a meeting between the DHSS and the Tobacco Advisory Council (TAC) in July 1974, the department put forward a proposal for a new voluntary agreement between the industry and government. Suggested measures included a 10 per cent levy on all cigarette promotions to fund government anti-smoking campaigns, a ban on coupons and gift schemes except for cigarettes with low tar yields, and starker health warnings on cigarette packets. When it came to sponsorship of sport by the tobacco industry, the department viewed this as a way around the television advertising ban. Owen asked manufacturers to inform the department if they intended to move into a “new area of sponsorship” and threatened that he was prepared to legislate on the matter if the situation worsened.^109^ A month later, in August, JP&S exacerbated the situation by producing an advert for the JPL which contained an illustration of cricketers in action, a packet of Player’s No. 6 alongside its logo, and copy which read “Player’s No. 6 backing British sports.”^110^ Owen was reportedly “shocked” upon seeing the advert, and the DHSS warned that if the League became “closely associated with a brand of cigarette” then all advertisements for it would have to carry a government health warning, and banners at televised matches would have to be covered. The department asked the TAC for reassurances that there would be no repeat of any such advert being issued in the future.^111^ In response, the advertisement was withdrawn and Imperial Tobacco made it known that they would not allow brand references to appear in advertisements for sponsored events. It also agreed that no pictures, even drawings, of athletes would feature in its future advertisements.^112^

In May 1975, however, the tobacco industry rejected Owen’s proposed voluntary measures.^113^ Frustrated by the snub, Owen and the DHSS began to look into whether tobacco, including its advertising and promotion, could be controlled under the 1968 Medicines Act as “a substance which is not itself a medicinal product but if used without proper safeguards, is capable of causing danger to the health of the community.”^114^ Legislation was prepared that would control the use of tobacco substitutes and additives in cigarettes, regulate tar and nicotine levels, force the industry to follow rules on the labelling of their products, and ban or control the advertising and promotion of tobacco products if voluntary agreements could not be reached.^115^

As these events were unfolding, an important figure became involved in the debate over the regulation of sponsorship: Denis Howell, Minister for Sport. Howell had played a significant role in the establishment of the Sports Council in 1965, becoming its first chairman (although he was not part of their enquiry into sponsorship). Throughout his political career, he was a consistent defender of the right of sports to accept sponsorship from the tobacco industry. In April 1974, at a lunch hosted by John Wilson (presumably the then director of Tetley Walker brewery, and chairman of Liverpool Football Club), Howell met the chairman of Imperial Tobacco, Tony Garrett. Garrett had previously been chairman of JP&S during the period in which the JPL was launched and he alerted Howell to the ongoing negotiations between the DHSS and TAC over tobacco sponsorship.^116^ Howell made it known to the DHSS that he was concerned about the effect that controls on sponsorship might have on sport. He took particular issue with the proposal to freeze cigarette manufacturers’ spending on sponsorship as part of any new voluntary code.^117^ It was agreed by the government’s Social Services Committee to allow Howell to lead negotiations with the industry over a voluntary agreement to cover sponsorship, as long as the DHSS were represented at any meetings with the industry.^118^

In producing the voluntary agreement, the tobacco industry – represented by Tony Garett – lobbied their inside sympathiser, Howell, to produce an early draft of the voluntary code.^119^ These discussions happened separately from those between the TAC and Howell and they were informal, taking place over lunches, indicating familiar avenues of corporate courtship. Howell also attended Imperial-sponsored events such as the John Player Grand Prix, perhaps because he had been given complimentary tickets.^120^ Garrett was eager to safeguard the company’s interests and he was at pains to have the difference between a ‘house’ name and a ‘brand’ name framed in the voluntary agreement.^121^ The DHSS took a “dim view” of the close relationship between Garrett and Howell, and this reflected growing tensions between the DHSS and the Minister for Sport over the regulation of sponsorship.^122^ One problematic issue was the proposed freeze on tobacco companies’ expenditure on sponsorship. Owen wanted this to be fixed, but Howell and Garrett wanted it to increase annually in order to stay in line with inflation.^123^ In September 1976, Owen was moved to a new position as Minister of State for Foreign and Common Affairs, and his proposed legislation on tobacco products fell by the wayside. Howell became free to draft the sponsorship agreement on his own, without close DHSS oversight, and the final version was produced in December 1977, more than two years after the process had begun. The final voluntary agreement bore close resemblance to the initial draft that the TAC had produced in 1975.^124^ The freeze on sponsorship expenditure proposed by the DHSS remained in place, but with allowances made for inflation as Howell and Imperial had wanted. Event names were to avoid any words with direct connotations to a brand, such as “filter,” “No. 6,” or “king size,” but, as JP&S had wanted, house names were permitted. Advertisements for a sponsored event were not permitted to include illustrations of cigarettes, the word cigarette, people pictured smoking or pictures of identifiable sports personalities. Finally, the code set out requirements regarding the number and size of signs at televised events. Signs could only display the name of the event and could not be situated in positions likely to linger within camera shot, such as on a scoreboard.^125^ All in all, the stipulations in the code represented a pragmatic outcome. On the one hand, the tobacco industry could say it was responsibly recognising that sports sponsorship had to be subject to certain controls, but on the other hand, by taking a central part in negotiations, Imperial tobacco managed to mitigate against controls being too all-encompassing, or restrictive.

The negotiations over the voluntary agreement governing sponsorship reflected a growing bullishness on the part of the tobacco industry. Instead of having controls imposed on it by figures such as the Postmaster General, as had happened with the television advertising ban in 1965, the industry successfully shaped those regarding sponsorship. This development should be understood in the context of a wider strategy which was adopted by leading international tobacco manufacturers in the spring of 1977. Garrett organised a secret meeting between Imperial Tobacco, Gallaher, Rothmans, British American Tobacco, Philip Morris, R.J. Reynolds, and Reemtsma at which it was decided that the industry would push back against tobacco control measures and refuse to cede concessions beyond certain agreed points.^126^

The industry’s increasing defensiveness was provoked by a changing political landscape. Formed in the UK in 1971, the pressure group Action on Smoking and Health (ASH) routinely called out what it saw as the hypocrisy of the tobacco industry’s advertising and promotions. In 1977, for instance, ASH wrote to Howell asking the minister to stop the sponsorship by a tobacco firm of the forthcoming series of cricket tests between England and Australia. Howell replied that “It is for the cricket authorities themselves to decide whether or not to accept sponsorship from tobacco firms.”^127^

Faced with growing anti-smoking activism, the tobacco industry used their influence to network with sportsmen and sports organising bodies. In 1984, for example, the British Medical Association (BMA) launched a campaign calling for all tobacco advertising and sponsorship to be banned, having labelled the voluntary agreements a “farce.”^128^ In response, the TAC, in what was the first in a series of leaflets which stressed the benefits of tobacco’s support for sport, highlighted sympathetic quotes from various individuals involved in British sport. This included Donald Carr, the Secretary for the TCCB, who explained that money from tobacco sponsorship was used towards running grassroots coaching schemes for young cricketers.^129^

The leaflets revealed the industry’s desire to protect the voluntary controls on tobacco sponsorship and represented an intensification of its lobbying activities. They forwarded a free-market defence of their right to use sport to market cigarettes. The TAC produced a leaflet in 1988, arguing that “Sporting bodies’ freedom of choice should be maintained.” The same leaflet contained the claim that many sports had benefited from generous tobacco sponsorship: “John Player has been acknowledged to have ‘saved’ cricket when it was struggling for survival as the national sport by the introduction of its Sunday League one-day series.”^130^ Such arguments found a receptive audience within the neo-liberally-minded Thatcher administrations, which included ministers sympathetic to the tobacco industry, such as Kenneth Clarke.^131^

By calling on supportive testimony from leading figures within British sport, and developing an alliance with the Minister for Sport the tobacco industry was able to strengthen the defence of its sponsorship activities as it introduced sympathetic stakeholders into the debate surrounding tobacco control measures. As the courting of Howell illustrates, these interests could help successfully dampen moves against tobacco promotion. The cultivation of sympathetic allies was a conscious strategy which was at the heart of the tobacco industry’s sponsorship activities. As laid out in its “Sponsorship Policy” during the mid-1970s, JP&S saw its involvement in sport as actively “helping to mobilise various ‘opinions’ in our favour.” It recognised the value of these opinions for the ability to influence government policy, especially given “the reduction of media available to us and the possible limitations imposed on us by politicians.”^132^ This picture complicates studies which have already shown how the relationship between the tobacco industry and the DHSS broke down in the 1970s.^133^ Working the channels of sports policy in Westminster, to all intents and purposes, allowed the tobacco industry to significantly forestall tighter controls. Subsequent voluntary agreements on sponsorship did not radically depart from the restrictions laid out in 1977 and tobacco sponsorship continued until the eventual ban on all tobacco advertising and promotion in 2002.

The precise reasons for JP&S’s decision to dissolve the JPL sponsorship deal in 1986 were kept relatively opaque in media reports, but they are easily surmised. Imperial had been taken over by the Hanson Trust in April and, although a spokesman denied there was a connection between this and the ending of JP&S’s involvement with cricket, there followed a period of company streamlining and asset stripping which resulted in more centralised oversight of advertising, promotions, and sponsorship by Imperial.^134^ The profile of smokers appeared to have changed too, making cricket and its gentlemanly associations less relevant for the product. Whereas in 1969, JP&S had identified the majority of match attendees as coming from within the ABC1 social classes (those with higher levels of disposable income), increasingly in the 1980s, smoking became concentrated amongst groups in the lower socio-economic brackets. Responding to these market research findings, Imperial decided to persist in sponsoring sports with more working-class profiles, such as snooker and rugby league.^135^ Furthermore, by the mid-1980s, there were wider signs that one-day cricket was losing its appeal. The *Financial Times* reported that only 134,000 people attended a JPL match during the 1985 season.^136^ With the TCCB’s decision to stage international test matches on Sundays, the BBC gave the League only 33 hours of television coverage in 1981.^137^

Although these cumulative circumstances led JP&S to dissolve its cricketing sponsorship, the response of its rival, Gallaher, to these changes was markedly different. Gallaher retained the naming rights for another long-running cricket competition, the Benson & Hedges Cup, between 1972-2002. The JPL was nevertheless one of the first major national sporting competitions to benefit from tobacco sponsorship. It provides exemplary insights, previously unexamined by historians, into how tobacco companies harnessed some considerable creatively in forging links to sport. Cricket provided its sponsors with invaluable television exposure and gave this increasingly taboo industry an opportunity to cement itself in the male consciousness. The partnership between tobacco and cricket thrived, in part, because of the historic connection between smoking and England’s summertime sport, while cricket itself needed a boost in the late 1960s to ward off the crisis of dwindling match attendance numbers.

**Figure 4:**
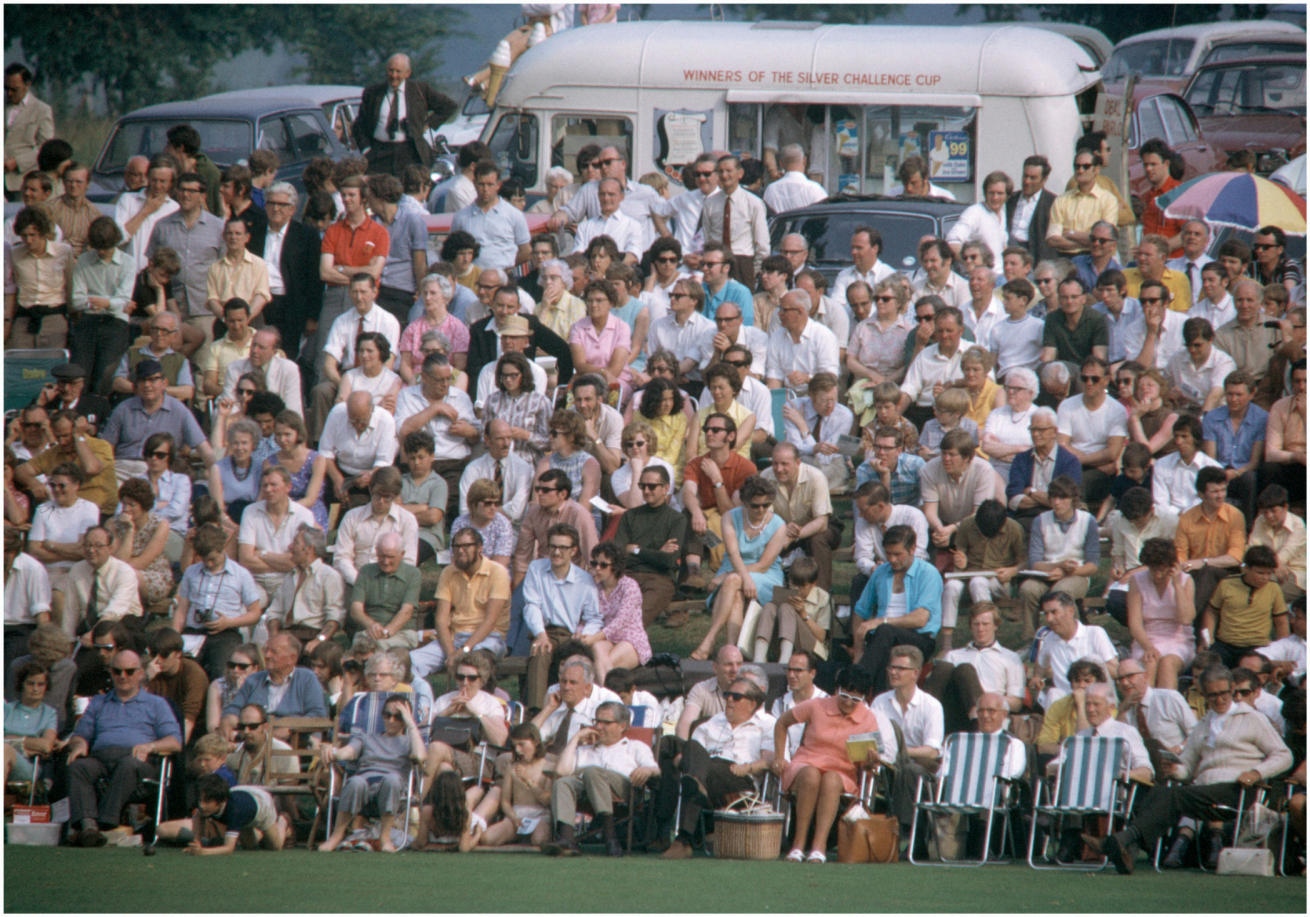
Crowds watching the John Player League match between Kent and Yorkshire at Mote Park, Maidstone, 4 July 1971. Credit: Patrick Eager/Getty Images.

JP&S, through its sponsorship of the new exciting format of the JPL, cleverly positioned itself within cricket as providing a public – even national – cultural good. JP&S routinely stated that it had “revitalised” cricket and given the sport a “shot in the arm.”^138^ Such self-congratulatory remarks formed the basis of the company’s defence of its sponsorship activities. One manager remarked that “[i]t is indisputable that without the financial backing of companies like Player’s some sports would suffer. Where would cricket be today without the one-day game?”^139^ In several ways, these claims were true. During the JPL’s first six years, annual attendances at League matches never dropped below 451,000 people and attendance figures reached as high as 625,000 during the 1975 season (see Fig. 4).^140^ During its first year, an average of 1.25 million viewers were said to have tuned into the JPL broadcasts on BBC2.^141^

Public awareness of the tobacco companies thus remained stubbornly high. In November 1975, National Opinion Poll Market Research Limited carried out a survey of 2,000 adult smokers and non-smokers who were asked to name any companies which sponsor sport, the arts, or entertainment. Of these, 758 respondents named JP&S, the highest total for any company, which compared to 302 who named Rothmans, and 267 who named Benson & Hedges.^142^ Although direct impacts on sales are notoriously hard to measure, JP&S likely benefitted from this publicity. Player’s No. 6 was Britain’s biggest selling cigarette between 1970 and 1978.^143^ Nevertheless, there were signs that public opinion was beginning to turn against smoking, despite the industry’s promotional efforts. Although the NOP survey identified those aged between 16 and 24 years as the age group most likely to identify a cigarette company as a sponsor of sport, only 36 per cent of this age group were “very much/somewhat in favour” of sponsorship by cigarette companies – the lowest approval rating for any of the age groups surveyed.^144^ Additionally, sales of cigarettes in the UK peaked in 1973 and thereafter began a gradual decline.^145^ These trends illustrate the wider cultural turn against smoking and tobacco promotion which then gathered pace, and which became increasingly apparent during the 1980s and beyond, as concerns grew over related health issues such as passive smoking.

Though public opinion eventually moved towards medical and moral outrage, sponsorship of the JPL earned JP&S an extra twenty years more public exposure than they would have otherwise been afforded, after public health fears about cigarette smoking escalated in the 1960s resulting in bans on advertising. JP&S saw in cricket the opportunity to associate its brands with excitement, glamour, and prestige. Cricket enabled tobacco, during its time of crisis, to remain covertly embedded in the national consciousness. Far from sitting on the side lines, these moves by JP&S were purposeful and strategic. They were strategies crafted to maintain, even extend, market share, when the health risks of smoking were becoming increasingly obvious and indefensible.

## Footnotes

1 T. Francis Jarman, letter to the editor *Guardian* (UK), 7 October 1970.

2 John L. Roberts, *Monitoring Tobacco-Sponsored Snooker on BBC TV 1985-86* (London, 1986); Simon Chapman, *Great Expectorations: Advertising and the Tobacco Industry* (London, 1986); Adhealth Limited, *Tobacco and the BBC: a Review of how BBC TV Promotes Cigarettes Through Tobacco-Sponsored Sport* (London, 1992).

3 Formula One motor racing teams sponsored by tobacco companies, however, managed to bypass some of the restrictions: see Timothy Dewhirst and Robert Sparks, “Intertextuality, Tobacco Sponsorship of Sports, and Adolescent Male Smoking Culture: a Selective Review of Tobacco Industry Documents,” *Journal of Sport and Social Issues*, 27 (2003), 372-398; Bruce Grant-Braham and John Britton, “Motor Racing, Tobacco Company Sponsorship, Barcodes and Alibi Marketing,” *Tobacco Control*, 21 (2001), 529-535.

4 For histories of smoking, see Virginia Berridge, *Demons: Our Changing Attitudes to Alcohol, Tobacco and Drugs* (Oxford, 2013); David Courtwright, “‘Carry on Smoking’: Public Relations and Advertising Strategies of American and British Tobacco Companies since 1950,” *Business History*, 47 (2005), 421-432; Rosemary Elliot, *Women and Smoking Since 1890* (New York, 2008); Matthew Hilton, *Smoking in British Popular Culture, 1800-2000* (Manchester, 2000); Penny Tinkler, *Smoke Signals: Women, Smoking and Visual Culture in Britain* (Oxford, 2006).

5 Timothy Chambers and Franco Sassi, “Unhealthy Sponsorship of Sport: Tougher and More Comprehensive Regulation is Long Overdue,” *British Medical Journal*, 367 (2019), doi:10.1136/bmj.l6718; Anne M. Lavack, “An Inside View of Tobacco Sports Sponsorship: an Historical Perspective,” *International Journal of Sports Marketing and Sponsorship*, 5 (2003), 14-37; N. Jennifer Rosenberg and Michael Siegel, “Use of Corporate Sponsorship as a Tobacco Marketing Tool: a Review of Tobacco Industry Sponsorship in the USA, 1995-99,” *Tobacco Control*, 10 (2001), 239-246.

6 Robert Proctor, *Golden Holocaust: Origins of the Cigarette Catastrophe and the Case for Abolition* (Berkeley, CA, 2011), 88-117. Some historical work on marketing strategies has been undertaken, but with no focus on sport.

7 Martin Polley, *Moving the Goalposts: A History of Sport and Society in Britain Since 1945* (London, 2003), 84; Dilwyn Porter, “British Sport Transformed: Sport, Business, and the Media since 1960,” in *Business in Britain in the Twentieth Century: Decline and Renaissance?* ed. Richard Coopey and Peter Lyth (Oxford, 2009), 330-350; Stephen Wagg, “‘Everyone Seemed to be “with it”’: Cricket, Politics and the Coming of the One-Day Game, 1940-1970,” *Sport in Society*, 16 (2013), 5-18.

8 Alongside other British tobacco manufacturing concerns, such as W.D. & H.O. Wills of Bristol, JP&S pioneered the use of mechanisation within the cigarette manufacturing process; see Hilton, *Smoking in British Popular Culture*, 83-84.

9 Virginia Berridge, *Marketing Health: Smoking and the Discourse of Public Health in Britain, 1945-2000* (Oxford, 2007), 21, 153-55.

10 Eadem, “Post-war Smoking Policy in the UK and the Redefinition of Public Health,” *Twentieth Century British History*, 14 (2003): 61-82, at 70-71.

11 Berridge, *Marketing Health*, 151-155.

12 Virginia Berridge, “Medicine and the Public: the 1962 Report of the Royal College of Physicians and the new public health,” *Bulletin of the History of Medicine*, 81 (2007), 286-311.

13 Virginia Berridge and Kelly Loughlin, “Smoking and the New Health Education in Britain 1950s-1970s,” *American Journal of Public Health*, 95 (2005), 956-964; Alex Mold, “‘Everybody Likes a Drink. Nobody Likes a Drunk’: Alcohol, Health Education and the Public in 1970s Britain,” *Social History of Medicine*, 30 (2017), 612-636.

14 “Albion Kick Smoking,” *Times* (London), 4 August 1984.

15 Blum, “Tobacco in Sport: an Endless Addiction?” *Tobacco Control*, 14 (2005), 1-2; Polley, *Moving the Goalposts*, 67-69.

16 John Horne and Gary Whannel, “Beer Sponsors Football: What Could go Wrong?” in *Sport, Beer, and Gender: Promotional Culture and Contemporary Social Life*, ed. Lawrence A. Wenner and Steven J. Jackson (New York, 2009): 55-74.

17 Allan Brandt, *Cigarette Century: The Rise, Fall, and Deadly Persistence of the Product that Defined America* (New York, 2008).

18 “Smokers v. Non-Smokers,” *Times* (London), 17 September 1884; “The English Cricketers in Australia,” *Times* (London), 6 May 1887.

19 “The Hit of the Century!” (advertisement), *The Illustrated London News*, 28 August 1897; “The Two Champion Players,” N.C.C. (Haddon’s) 1956-59 Guard Book, John Player Advertising Archive (hereafter: JPAA), Nottingham. The second advertisement was originally published in 1897 and then republished in 1957.

20 Barbara Forey, Jan Hamling, Peter Lee and Nicholas Wald, *International Smoking Statistics: A Collection of Historical Data from 30 Economically Developed Countries* (Oxford, 2002), 649.

21 Monopolies Commission, *Report on the Supply of Cigarettes and Tobacco and of Cigarette and Tobacco Machinery* (London, 1961), 39.

22 “Great Player’s” (advertisement), *Daily Mirror* (London), July 15, 1926; “Player’s Please” counter card, 1934, 2001-33/559a, Nottingham City Museums and Galleries (hereafter: NCMG); “Player’s Please” counter card, 1934, 2001-33/556, NCMG.

23 ”Army Club Cigarettes” (advertisement), *Daily Mirror* (London), 27 May 1926; “Nothing Throaty About Sarony” (advertisement), *Daily Mirror* (London), 13 June 1928.

24 “Len Hutton”, 1946, C-676270, British Film Institute (hereafter BFI); “Denis Compton”, 1949, C-755920, BFI; “Godfrey Evans”, 1952, C-755895, BFI.

25 “Len Hutton Right on the Ball!” (advertisement), *Daily Mirror* (London), 16 June 1954; “Smith Empire Watches” (advertisement), *Daily Mirror* (London), 16 September 1954; “When I Smoke – I’m Particular,” *Daily Mirror* (London), 13 July 1952.

26 Daniel O’Neill, “‘People Love Player’s’: Cigarette Advertising and the Teenage Consumer in Post-war Britain,” *Twentieth Century British History* 28, no.3 (2017), 414-439.

27 Wagg, “‘Everyone Seemed to be “with it”’,” 10-13.

28 Brian Glanville, “No Easy Way to Brighter Cricket,” *Sunday Times* (London), 29 January 1961.

29 Wagg, “‘Everyone Seemed to be “with it”‘,” 10-13.

30 Ibid.

31 Richard Doll and A. Bradford Hill, “Smoking and Carcinoma of the Lung,” *British Medical Journal*, 30 (1950), 739-748.

32 “Heavy Smoking and Cancer,” *Times* (London), 13 February 1954.

33 “Effects of Smoking on Health,” *Financial Times* (London), 13 February 1954.

34 Research Services Limited, “Cigarette Smoking Motivation Study,” 6 March 1956, 41, HAT 50/1/137/2/3/1, J. Walter Thompson client account files, History of Advertising Trust (hereafter HAT).

35 Hilton, *Smoking in British Popular Culture*, 202-204; Berridge, “Medicine and the Public,”, 299-301.

36 Royal College of Physicians, *Smoking and Health* (London, 1962), 54.

37 “Notes on Guidance on Television Advertisements for Cigarettes and Hand-Rolling Tobaccos,” 10 August 1962, HAT/50/1/137/2/2/2, J. Walter Thompson client account files, HAT.

38 “The New Code for Cigarette Advertising”, *Advertising Quarterly*, 45 (1975), 16.

39 *Statistical Review of Press and TV Advertising*, 33 (January–March, 1965), 32, 42; ibid. (April–June, 1965), 4.

40 Business of the House, *Parliamentary Debates*, Commons, 16 January 1964, 5th series, vol. 687, col. 412-3; ibid., 4 February 1965, 5th series, vol. 705, col. 1291.

41 John Horam, “Smokers Pay for the NHS and the Motorways as Well,” *Financial Times* (London), 27 March 1965.

42 Conrad Jameson Associates, “House Image of Player’s for Smokers Under 30,” April 1965, 1-3, uncatalogued, JPAA.

43 Ray Spencer, “Tobacco Companies Play with Brands to Beat Chancellor’s Regulator,” *Financial Times* (London), 21 July 1969.

44 “Player’s Join Gift Coupon Cigarettes War,” *Financial Times* (London), 19 November 1965.

45 “About the Firm No. 11: Marketing and Sales, Part 1,” *Navy Cuttings* (Nottingham), June 1966, 43-44.

46 “Introductory Speech by Mr G.C. Kent,” 1968, Box D15a, JPAA.

47 Ogilvy and Mather to Trevor King (No. 6 brand manager), 1 December 1967, Box D38, JPAA; Phillip Vickers, “Proposals for Two Promotions for Player’s No. 6 in 1968,” 28 December 1967, Box D38, JPAA.

48 Roger Eglin, “Pay up! Pay up! and Play the Game!” *Observer* (UK), 6 September 1970.

49 G.D. Campbell, “1968/69 Player’s No. 6 Promotional Activity,” 4 April 1968, Box D38, JPAA.

50 M.J. Vigor to multiple, 5 November 1969, Box D16a, JPAA.

51 John Bromley, “Sportlight,” *Daily Mirror* (London), 27 March 1963.

52 “Dexter Helps,” *Daily Mail* (London), 27 March 1963; Alex Bannister, “Cricket Showed the Way,” *Daily Mail* (London), 22 December 1973.

53 “Sunday Games will be Televised,” *Daily Mail* (London), 22 February 1965.

54 “Sir Donald Denies Test Match ‘Rigging’,” *Times* (London), 7 February 1963.

55 Michael Parkinson, “Tilting for the Cavaliers,” *Sunday Times* (London), 6 April 1969.

56 “Player’s No. 6 County Cricket Championship,” 21 August 1968, Box D16a, JPAA.

57 “Time a Hatchet was Buried,” *Guardian* (UK), 21 December 1968.

58 Billy Griffith, “Richer on Sundays,” *Sunday Times* (London), 20 April 1969.

59 Bagenal Harvey, “Cricket,” *Sunday Times* (London), 27 April 1969.

60 “A Startling Letter,” *Sunday Times* (London), 27 April 1969.

61 “Cavaliers to Continue,” *Guardian* (UK), 20 December 1969.

62 “Sponsorship Policy,” 2 February 1975, Box D17, JPAA.

63 “Player’s No. 6 County Cricket Championship,” 21 August 1968, Box D16a, JPAA.

64 K.A. Heffernan, “Player’s County League 1969,” 12 November 1969, Box D16a, JPAA.

65 P.C. Taylor, “Budgets – Brand Group Contributions,” 14 October 1975, Box D17, JPAA; Peter Taylor, “Estimated Television Air Time for Special Events,” 6 August 1976, Box D15b, JPAA.

66 Peter Taylor, “Reverse Priority List: Order of Rejection,” 26 May 1976, Box D33, JPAA.

67 P.C. Taylor, “Budgets – Brand Group Contributions,” 14 October 1975, Box D17, JPAA.

68 “Current Situation – John Player Team Lotus,” 27 November 1975, Box D24, JPAA.

69 “Sponsorship Policy,” 2 February 1975, Box D17, JPAA.

70 Ibid.

71 Jim Shaw, “Player’s Promotional Policy: ‘To be Where the Action is’,” *Player’s Post* (Nottingham), September 1969.

72 “‘Sponsorship Policy’,” 2 February 1975, Box D17, JPAA.

73 “‘Sponsorship Policy’,” 2 February 1975, Box D17, JPAA.

74 “Player’s No. 6 National Promotions Y/E 31.10.69,” 11 November 1968, Box D38, JPAA.

75 Cigarette Smoking (Gift Schemes and Advertising), Parliamentary Debates, Commons, 23 October 1967, 5th series, vol. 751, cols. 1328-1329.

76 J.P. Shaw, “Player’s County League,” 3 September 1969, Box D16a, JPAA.

77 J.G. Seager, “Player’s County League,” 10 September 1969, Box D16a, JPAA.

78 “Player’s County League,” 1 October 1969, Box D16a, JPAA.

79 M.J. Vigor, “Player’s No. 6 - Player’s County League,” 18 April 1969, Box D16a, JPAA.

80 The National Archives (hereafter: TNA), “Note 3 Cigarette Advertising,” December 1975, MoH (Ministry of Health and its successors, hereafter: MoH) 154/1482.

81 “Brief Clips of Barry Richards and Gordon Greenidge, Hampshire v Derbyshire JPL, 1975,” YouTube, <www.youtube.com/watch?v=LB1xYgpGBXA>, accessed 28 June 2021.

82 “‘Sponsorship Policy’,” 2 February 1975, Box D17, JPAA.

83 D.L. Way, “SEU Strategy/Objectives,” 18 December 1975, Box D22, JPAA.

84 “Special Events Unit Advertising Grant Breakdown 1975/76,” 14 October 1975, Box D17, JPAA.

85 “Cigarettes (Warning Notices),” *Parliamentary Debates*, Commons, 24 June 1971, vol. 819, col.340-4w.

86 “The John Player League” (advertisement), *Sunday Times Magazine* (London), 20 August 1972, 2-3; “The Edinburgh Tattoo” (advertisement), *Sunday Times Magazine* (London), 30 July 1972, 2-3; “Wimbledon Fortnight” (advertisement), *Sunday Times Magazine* (London), 17 June 1973, 32-33; “The Southport Flower Show” (advertisement) *Sunday Times Magazine*, 18 August 1974, 5-4.

87 “Sundays are Rundays in the John Player League” (advertisement), *Sunday Times Magazine* (London), 20 May 1973, 4-5; “Kenwood Open Air Concerts” (advertisement), *Sunday Times Magazine* (London), 23 June 1974, 8-9.

88 “‘Bringing you the Best’ – at 240 Oxford Street,” *Player’s Post* (Nottingham), 31 August 1972, 4-5.

89 “The John Player Information Bureau” (advertisement), *Sunday Times Magazine* (London), 18 February 1973, 64-65.

90 T.C.H. King, “Player’s No. 6: Future Promotional Policy,” 18 July 1968, Box D38, JPAA.

91 “Sundays are Rundays,” 4-5.

92 Other tobacco companies also engaged in this branding sleight of hand, but in an even more brazen manner. From 1972, Gallaher sponsored another one-day cricket tournament, the ‘Benson & Hedges Cup’. ‘Benson & Hedges’ was not strictly a company name, as the manufacturer had been taken over by Gallaher in 1955. Rather, ‘Benson & Hedges’ was an umbrella name, or a ‘house’ name, under which a series of brands was launched, including Silk Cut, Sovereign, and Special Filter.

93 “Player’s No. 6 County Cricket Championship,” 21 August 1968, Box D16a, JPAA.

94 G.K. Hadfield, “Player’s No. 6- Player’s County League,” 13 February 1969, Box D16a, JPAA.

95 Jim Shaw, “Player’s Promotional Policy: ‘To be where the Action is’,” *Player’s Post* (Nottingham), September 1969.

96 M.J. Vigor, “Player’s No. 6: Player’s County League,” 18 April 1969, Box D16a, JPAA.

97 “Lady Demonstrators” (advertisement), *Sunday Times* (London), 30 March 1969; “Lady Demonstrators” (advertisement), *Sunday Times* (London), 28 September 1969; “Lady Demonstrator” (advertisement), *Sunday Times* (London), 7 March 1971.

98 G.K. Hadfield, “Player’s No. 6 – Player’s County League,” 13 February 1969, Box D16a, JPAA.

99 K.A. Heffernan, “Player’s County League 1969,” 12 November 1969, Box D16a, JPAA.

100 K.A. Heffernan, “Player’s County League 1969,” 12 November 1969, Box D16a, JPAA.

101 Robin Marlar, “Coughin’ Cup Traps Kids,” *Sunday Times* (London), 23 May 1976.

102 TNA, H. B. Grice to W. G. Hammerton, 21 June 1976, MoH 154-1014.

103 “Player’s No. 6 ‘Open the Box’ Show,” 29 January 1969, Box D16b, JPAA; “General Note for all Unit Heads Over 2 Days,” n.d., Box D41, JPAA.

104 “Player’s No. 6 ‘Open the Box’ Show,” 29 January 1969, Box D16b, JPAA.

105 G. D. Campbell to G. K. Hadfield, 14 January 1969, Box D16b, JPAA.

106 Sports Council, *An Inquiry into Sponsorship: An Abridged Version of the Report Submitted to the Advisory Sports Council in 1971 by a Working Party set up to Look into all Aspects of Sponsorship of Sport* (London, 1972), 15-16.

107 TNA, Letter to F.M. Bell, 18 October 1971, MoH 154/590.

108 TNA, J. Bolitho, “Sponsorship by Tobacco Manufacturers,” 8 October 1973, MoH 154/590.

109 TNA, “Parliamentary Secretary (Health) Meeting Note, 58/74,” 17 July 1974, MoH 154/590.

110 “John Player League Cricket” (advertisement), *Daily Mirror* (London), 17 August 1974.

111 TNA, W.G. Hammerton to H.B. Gries, 21 August 1974, MoH 154/590.

112 TNA, S. Fox to A.C. Spencer, 24 September 1974, Box D29, JPAA.

113 “Notes Concerning Discussions with DHSS/Tobacco Advertising,” 1 July 1975, Box D42, JPAA.

114 TNA, “Social Services Committee, Use of the Medicines Act of 1968 to Control the Sale and Advertising of Tobacco Products,” July 1975, AT 60/16.

115 “Smoking and the Medicines Act,” 30 July 1975, AT 60/16.

116 TNA, R.A. Garrett to Denis Howell, 16 May 1975, AT 60/16.

117 TNA, Letter from J.J. Rendell, 3 July 1975, MoH 154/590; TNA, “Minister of State (Health) Meeting Note No. 89/75,” 9 July 1975, MoH 154/590.

118 TNA, “Sports Sponsorship by Tobacco Firms,” 7 October 1975, MoH 154/590.

119 TNA, R.A. Garrett to Denis Howell, 16 May 1975, AT 60/16; TNA, R.A. Garrett to Denis Howell, 22 July 1975, AT 60/16.

120 TNA, Denis Howell to D.H. Delamont, 25 March 1975, AT 60/16.

121 TNA, “Sports Sponsorship by the Tobacco Industry,” 24 February 1976, AT 60/17.

122 TNA, “Tobacco Sponsorship,” 5 March 1976, AT 60/17.

123 TNA, “Mr. Lillywhite,” 7 April 1976, MoH 154/590; “Sports Sponsorship by the Tobacco Industry: Proposed Voluntary Code of Practice,” 17 June 1976, AT 60/17.

124 TNA, “Draft Code Governing Recognition of the Sponsorship of Sporting and Other Events by Cigarette Manufacturers,” 27 March 1975, MoH 154/590.

125 TNA, “Agreement on Sponsorship of Sport by the Tobacco Industry,” 15 December 1975, MoH 154/1481; TNA, “Code of Practice Governing the Sponsorship of Sporting Events by Cigarette Manufacturers in the UK,” 15 December 1977, MoH 154/1481.

126 Neil Francey and Simon Chapman, “‘Operation Berkshire’: The International Tobacco Companies’ Conspiracy,” *British Medical Journal*, 321 (2000), 371-374.

127 TNA, Denis Howell to Mr Daube, 16 February 1977, AT 60/160.

128 Nicholas Timmins, “Doctors Call for End to All Advertising by Tobacco Companies,” *Times*, 17 October 1984.

129 “Tobacco Advisory Council, *Sports Sponsorship by Tobacco Companies: a Case for the Existing Voluntary System* (April 1985),” Industry Documents Library, University of California San Francisco, <www.industrydocuments.ucsf.edu/docs/#id=hrby0197>, accessed 28 June 2021.

130 “Tobacco Advisory Council, *Sports Sponsorship by Tobacco Companies: a Working Partnership* (August 1988),” Industry Documents Library, University of California San Francisco, <www.industrydocuments.ucsf.edu/docs/#id=nklj0101>, accessed 28 June 2021.

131 Berridge, *Marketing Health*, 243.

132 “Sponsorship Policy,” 2 February 1975, Box D17, JPAA.

133 Berridge, *Marketing Health*, 21, 153-55.

134 Imperial Tobacco Group, *The Imperial Story, 1901-2001: Celebrating One Hundred Years* (Bristol, 2001), 58; “Players Still,” *Financial Times* (London), 3 July 1986.

135 K.A. Heffernan, “Player’s County League 1969,” 12 November 1969, Box D16a, JPAA; Berridge, *Marketing Health*, 241.

136 Philip Coggan, “Time to Hit Clear of the Cricketing Trap,” *Financial Times* (London), 25 April 1987.

137 “Sport’s Real Winners,” *Daily Mirror* (London), 5 August 1982.

138 “The John Player League” (advertisement), *Sunday Times Magazine* (London), 11 June, 1972, 8-9; “Player’s County League is Shot in Arm for Cricket,” *Player’s Post* (Nottingham), June 1969, 9.

139 “Sponsorship the Reasons Why,” n.d., Box D15b, JPAA.

140 “John Player League,” n.d., Box D17, JPAA; P.C. Taylor, “Budgets – Brand Group Contributions,” 14 October 1975, Box D17, JPAA.

141 It should be noted, however, that these figures were paltry compared to the average of 4.5 million viewers achieved by the Cavaliers on ITV in the same year. K.A. Heffernan, “Player’s County League 1969,” 12 November 1969, Box D16a, JPAA.

142 Of the 758 people naming JP&S as a sponsor, 212 recognised that they sponsored cricket. M.R. Department (I.T.L.), “Survey of Public Awareness and Attitudes to Sponsorship,” 30 January 1976, Box D14a, JPAA.

143 Maxwell International Estimates, “How the Brands Ranked,” *World Tobacco*, June 1972, 57; ibid., July 1979, 68.

144 M.R. Department (I.T.L.), “Survey of Public Awareness and Attitudes to Sponsorship,” 30 January 1976, Box D14a, JPAA.

145 Forey, Hamling, Lee and Wald, *International Smoking Statistics*, 650.

